# Resting-state BOLD temporal variability in sensorimotor and salience networks underlies trait emotional intelligence and explains differences in emotion regulation strategies

**DOI:** 10.1038/s41598-022-19477-x

**Published:** 2022-09-07

**Authors:** Federico Zanella, Bianca Monachesi, Alessandro Grecucci

**Affiliations:** 1 Clinical and Affective Neuroscience Lab - Cli.A.N Lab, Department of Psychology and Cognitive Science, University of Trento, Trento, Italy; 2 Centre for Medical Sciences, CISMed, University of Trento, Trento, Italy

**Keywords:** Neuroscience, Emotion, Neural circuits

## Abstract

A converging body of behavioural findings supports the hypothesis that the dispositional use of emotion regulation (ER) strategies depends on trait emotional intelligence (trait EI) levels. Unfortunately, neuroscientific investigations of such relationship are missing. To fill this gap, we analysed trait measures and resting state data from 79 healthy participants to investigate whether trait EI and ER processes are associated to similar neural circuits. An unsupervised machine learning approach (independent component analysis) was used to decompose resting-sate functional networks and to assess whether they predict trait EI and specific ER strategies. Individual differences results showed that high trait EI significantly predicts and negatively correlates with the frequency of use of typical dysfunctional ER strategies. Crucially, we observed that an increased BOLD temporal variability within sensorimotor and salience networks was associated with both high trait EI and the frequency of use of cognitive reappraisal. By contrast, a decreased variability in salience network was associated with the use of suppression. These findings support the tight connection between trait EI and individual tendency to use functional ER strategies, and provide the first evidence that modulations of BOLD temporal variability in specific brain networks may be pivotal in explaining this relationship.

## Introduction

Emotion regulation (ER) is employed to modify current emotional states and adaptively respond to the environment^1–4^. Not surprisingly, difficulties in ER are involved in compromised well-being and mental health^5,6^. Namely, the selection of specific strategies and the excessive or rigid usage of them has been associated with negative outcomes^7–9^ contributing to a general distinction between dysfunctional and functional strategies. For example, some studies reported that suppression strategy (i.e., the inhibition of the expressive reactions to the emotional events) fails in engendering subjective relief after experiencing negative emotions, and it is associated with costs in terms of physiological, cognitive and social functioning^5,10,11^, as well as with decreased well-being^12^ and several psychopathologies (e.g.^13,14^). Differently, reappraisal strategy (i.e., the ability of cognitively changing the impact of the emotional event) has been generally considered an adaptive strategy, which is associated with healthiness and personal satisfaction^5^ improving the affective state^15^ and successfully modulating the emotion-related peripheral physiological indexes^16^ and the neural activity^17^.

The simplistic distinction between functional and dysfunctional strategies, however, risks to conceal the role of individual differences in selecting and recurrently using regulation strategies^18^. What leads someone to use one given strategy or to use it more often than others? Even though individuals’ dispositions may be crucial to comprehend the variability behind the selection and usage of emotion regulation strategies^19^, investigations in this sense are still scant^18,20^. Only recently, an interesting line of research suggests a link between ER processes and the construct of emotional intelligence (EI^18^), with some authors suggesting a theoretical framework to explain such a relationship (e.g.^21^). In literature, EI can be operationalized both as a set of cognitive abilities, which allows the individual to perceive, reason about, and manage emotional information (ability EI—what individuals are able to do), and as a set of personal dispositions related to emotional events (trait EI—what individuals typically do^18^). Especially trait EI has been suggested to play a critical role in the ER processes^19^, since personality traits may shape individual preferences, interpersonal behaviours, and intents, which are all likely to influence the choice of ER strategy (e.g.^22^). In line with that, behavioural studies reported that individuals with high levels of trait EI tend to use contextually appropriate coping strategies, whereas individuals with low levels of EI adopted non-adaptive ones^18,23–25^. Differently, similar levels of ability EI may result in adaptive as well as deviant responses^22,26^, and there is evidence that the ability EI fails in predicting specific emotion regulation processes^27,28^, or when it does, it has to be coupled with trait EI to comprehend the beneficial consequences of the adaptive response^29^. The measure of traits EI, then, not only represent an individual’s predisposition measure itself, but it also seems promising to explain how ER processes are selected. However, the relationship between trait EI and specific ER strategies, the nature and the extent of their point of connection remain unclear, especially in neural terms. To fill this gap, the present study is aimed to investigate whether the link between trait EI and ER relies on a shared neural substrate, provided more in deep their relationship in terms of self-reported measures.

So far, the few functional^30,31^ and structural^32^ neuroimaging studies dealing with trait EI seem to support at least a partial neural overlap with findings from neuroimaging studies of ER^33–36^. Indeed, both EI^30,31^ and ER processes^33–38^ rely on task-related activity in brain areas subserving emotion processing (limbic system) and higher executive functioning (prefrontal regions). Similarly, from a structural neuroimaging perspective, it has been shown that grey matter alterations in fronto-limbic brain areas correlates with specific ER processes^39,40^ and traits EI^32^.

Besides structural and task-related functional evidence, some authors relied on resting-state functional connectivity (RS-FC) to understand the neural signature of ER and EI. RS-FC is a valuable source to understand the neural mechanisms behind several psychological states^20,41,42^, as well as emotional processes^17,33^. In addition, altered functional connectivity seems to be associated with many psychopathologies^43,44^ as ER^5^ and EI also are^45^. Evidence from resting-state studies showed that higher trait EI scores was associated with the amplitude of low-frequency fluctuations (ALFFs) in networks related to social emotion processing and cognitive control (Ref.^46^, but see^47^ for null results). Moreover, Takeuchi et al.^48^, showed that trait EI was associated with components of social cognition and somatic marker circuitry neural networks. They found the trait EI score positively correlated with resting-state activity between medial prefrontal cortex (mPFC) and the precuneus, the intracalcarine cortex, and between anterior insula (aIC) and the right portion of the dorsolateral prefrontal cortex (dlPFC). In ER context, Pérez et al.^49^ reported a negative correlation between the connectivity of the right basolateral amygdala, the left insula, and the supplementary motor cortex (SMA) with the frequency of use of cognitive reappraisal, and a positive correlation between emotion suppression strategy and the activity of the right basolateral amygdala, the dorsal anterior cingulate (dACC), the supplementary motor cortex (SMA), and in the left medial portion of the amygdala. In contrast, Uchida et al.^50^ reported a negative correlation between functional connectivity in the amygdala and medial prefrontal cortex (mPFC) with the success of applying the cognitive reappraisal strategy. It is important to note also that Dörfel et al.^51^, failed to replicate and extend both the previously mentioned studies. Notably, the inconsistencies in ER and EI studies, may derive from some methodological issues.

Indeed, the majority of studies used massive univariate approaches, and a priori selected regions^30,36,46,49,51^. Widely distributed processes, such as ER or EI, may be better captured using multivariate approaches and a network perspective^52^. For this reason, in the present study, we adopted a whole brain data-driven approach (independent component analysis, ICA) that allows us to identify the variations of BOLD signal activity over time within major brain networks rather than considering signals from a priori decided regions of interest. ICA, being a blind source separation method, is an unsupervised machine learning approach able to identify non-overlapping independent neural circuits^53^. Such independent circuits represent meaningful naturally separated circuits that bypass anatomically and histologically based regions, and have the advantage of reducing brain complexity into low dimensional spaces^52^. The BOLD temporal variability (SD BOLD) for every resting-state network was used to predict trait EI and ER strategies. BOLD variability is defined as a fluctuation in neural activity over time, and an increment in this measure reflects greater functional network complexity and is associated with more effective information integration^54–56^. According to Moreira et al.^57^, this feature is particularly relevant for psychological phenomena that develop over time, such as ER^58,59^, or affective states^60^ and represents an index of the degree of cognitive flexibility^61^.

Building on the above considerations, the present study is aimed to support and extend previous findings on the role of trait EI in determining ER strategies, and to disclose which overlapping resting-state networks may subserve this relationship. We hypothesize that the level of trait EI (measured by the TeiQue-SF questionnaire^62^) is associated with the use of different ER processes, assessed using two different scales, ERQ^12^ and CERQ^63^. These scales are largely used together in literature since they allow to cover a wide range of emotion regulation strategies. Namely, we expect that the higher the trait EI and/or subscales scores, the higher the usage of adaptive ER strategies, and/or the lesser the usage of non-adaptive ER strategies. Then, we delineate the networks underlying EI using an ICA approach on the resting-state functional connectivity, and we assess whether these networks may be associated with the use of different ER strategies. We expect to find that the activity in regions responsible for the processing and control of socio-emotional information (e.g., insula, frontal and parietal regions^64^) underly both the individual differences in EI (as measured by TeiQueSF), and the ER usage (measured by ERQ and CERQ).

## Results

### Trait measures

The total EI trait index is negatively associated (negative *β* values) with the frequency of use of the following strategies: Suppression (ERQ) (*F*(1,78) = 9.705, *R*^2^ = 0.11, *r* =  − 0.335; *p* = 0.003; *β* =  − 0.335; *p*FDR = 0.022) and Self-blame (CERQ) (*F*(1,78) = 7.867, *R*^2^ = 0.093, *r* =  − 0.304 *p* = 0.006; *β* =  − 0.304, *p*FDR = 0.022). For what concerns the four TEIQue-SF factors, we firstly confirmed that there was no multicollinearity between these predictors, as the Pearson’s correlation coefficient (less than 0.9^65^) and the Variance Inflation Factor (VIF, less than 10^66^) showed (see Table 1). Then, regression results showed that Emotionality (*β* = − 0.305, *p*FDR = 0.04), Well-being (*β* =  − 0.255, *p*FDR = 0.01) and female gender (*β* =  − 0.255, *p*FDR = 0.04) were associated with Suppression (ERQ) (*F*(4,75) = 4.908 R^2^ = 0.21; *p* = 0.001); Self-control (*β* = -0.366, *p*FDR = 0.01), Emotionality (*β* = 0.323, *p*FDR = 0.01), and Well-being (*β* =  − 0.254, *p*FDR = 0.03) were associated with Rumination (CERQ) (*F*(4,75) = 5.332, *R*^2^ = 0.28, *p* < 0.005); Finally, Self-control (*β* =  − 0.285, *p*FDR = 0.05), and Well-being (*β* =  − 0.336, *p*FDR = 0.02) were associated with Catastrophizing (CERQ) (*F*(4,75) = 4.258, *R*^2^ = 0.21, *p* < 0.001). To conclude, as can be noted from the negative *β* values of the predictors, the lower is the EI, the higher is the individual tendency to use non-adaptive ER regulation strategies such as Rumination, Self-blame, Catastrophizing, Suppression.

**Table 1:** Multi-collinearity tests (Pearson’s correlation and VIF) among the four TEIQue-SF total index and factors.

TEIQue_SF Pearsons’ correlations						Multicollinearity diagnostic
	Total EI	Self-control	Emotionality	Sociability	Well-being	VIF
Total EI	1					–
Self-control	0.629**	1				1.167
Emotionality	0.696**	0.280*	1			1.189
Sociability	0.482**	0.149	0.305**	1		1.128
Well-being	0.720**	0.308**	0.220	0.203	1	1.115

**p < 0.005.

### Functional connectivity results

Multiple Regression analyses (stepwise) showed that the BOLD variability of IC20 (*β* = 0.302, *p*FDR = 0.008) significantly predicts total EI index (*F*(21,76) = 0.07, *R*^2^ = 0.08, *p* = 0.008); IC18 (*β* = 0.360, *p*FDR = 0.04) and IC16 (*β* = 0.239, *p*FDR = 0.05) predict Sociability (*F*(4,74) = 6.02, *R*^2^ = 0.250, *p* < 0.001); Finally, IC20 (*β* = 0.380, *p*FDR = 0.03), IC1 (*β* = − 0.309, pFDR = 0.01), and IC7 (*β* = 0.234, *p*FDR = 0.03) predict Well-being (*F*(3,75) = 5.734, *R*^2^ = 0.18, p = 0.001). To summarise, the ICs associated with the total index and the subscale of the TEIQue-SF questionnaire were the IC1, IC7, IC16, IC18, and IC20. These IC’s include clusters of cortical and subcortical regions at a cluster significance level *p* < 0.05 (*p*FDR corrected) and voxel significance *p* < 0.001 (*p*FDR corrected). Based on CONN’s correlational spatial match to template approach, the identified ICs are attributable to known resting state networks: IC20 (*r* = 0.46) and IC16 (*r* = 0.36) = sensorimotor network; IC1 (*r* = 0.49) = cerebellar network; IC18 (*r* = 0.58) = visual network, and IC7 (*r* = 0.10) = salience network.

Among the IC’s associated with trait EI, the BOLD variability of IC20 (*β* = 0.492, *p*FDR = 0.005) was also associated with cognitive reappraisal (ERQ) (*F*(6,72) = 3.151, *R*^2^ = 0.21, *p* = 0.008). The BOLD variability of IC20 (*β* = 0.345, *p*FDR = 0.01), IC16 (*β* =  − 0.275, *p*FDR = 0.02) and IC7 (*β* = 0.299*, p*FDR = 0.01), was also associated with Positive Reappraisal (CERQ) (*F*(7,71) = 3.544, *R*^2^ = 0.22, *p* = 0.004). Finally, the BOLD variability of IC7 (*β* =  − 0.335, *p*FDR = 0.006) and female gender (β =  − 0.360, pFDR = 0.006) was associated with Suppression (ERQ) (*F*(6,72) = 4.271, *R*^2^ = 0.26, *p* = 0.001) (see Table 2).

**Table 2:** Summary of the main neural results.

ICs	ER and trait EI	*β*	pFDR
IC20 (sensorymotor)	Cognitive reappraisal (ERQ)	0.492	0.005
	Positive reappraisal (CERQ)	0.345	0.01
	Total EI (TeiQue-SF)	0.302	0.008
	Wellbeing (TeiQue-SF)	0.380	0.03
IC16 (sensorymotor)	Positive reappraisal (CERQ)	− 0.275	0.02
	Sociability (TeiQue-SF)	0.239	0.05
IC7 (salience)	Suppression (ERQ)	− 0.335	0.006
	Positive reappraisal (CERQ)	0.299	0.01
	Wellbeing (TeiQue-SF)	0.234	0.03

Beta (*β*) and corrected p-value (*p*FDR) for the significant relationships between the BOLD temporal variability in the ICs (IC20, IC16, IC7) and both ER strategies and EI subscales.

To sum up, the higher is the BOLD variability the higher is the frequency of use of cognitive reappraisal and positive reappraisal (with the exception of IC16). Whereas, the lower is the BOLD variability the higher is the frequency of use of Suppression.

**Figure 1:**
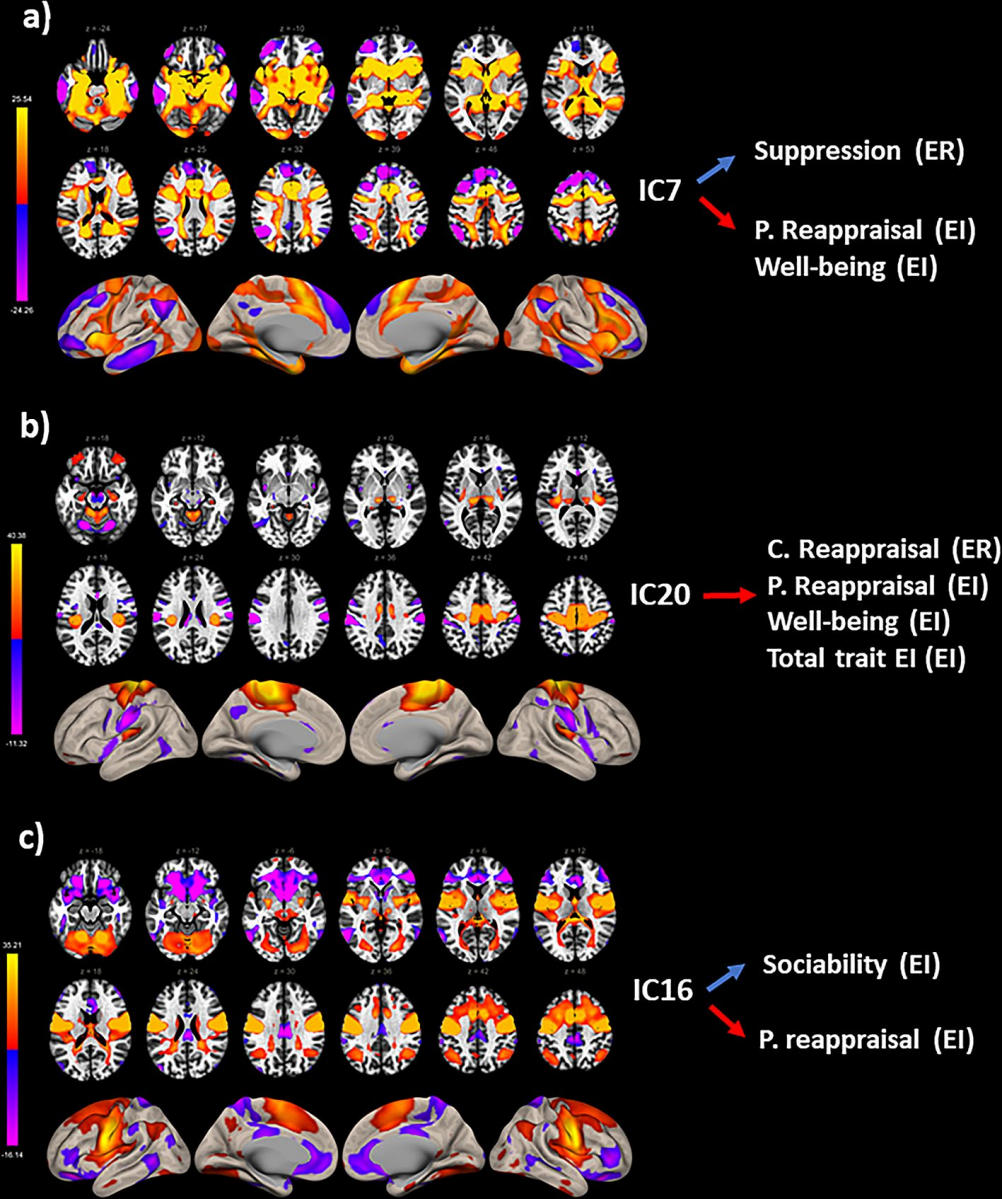
Representation of the ICs shared by trait EI and ER. (**a**) IC7; (**b**) IC20 and (**c**) IC16. Left of the panel: 2D (above) and 3D inflated (below) brain model for the three ICs (cluster significance *p*FDR < 0.05 and voxel significance pFDR < 0.001). Colour bar represents positive *t-*values in orange and negative *t-*values in blue. Right of the panel: Relation between the ICs and the subscales of trait EI and ER questionnaires. In red, the positive relation; In blue, the negative relation. *C. Reappraisal* cognitive reappraisal, *P. Reappraisal* positive reappraisal.

The brain networks identified by the IC7, IC20, and IC16 include clusters of cortical and subcortical regions (see Table 3 for details) at a cluster significance level *p* < 0.05 (*p*FDR corrected) and voxel significance *p* < 0.001 (pFDR corrected) (see Fig. 1).

**Table 3:** Regions identified in the independent component IC7, IC20, and IC16.

IC	Peak	ROI (Harvard, Oxford Atlas)	p-valueFDR
IC7	+ 02 + 12 + 46	L/R Amygdala	< 0.005
	+ 02 + 12 + 46	Cingulate gyrus	< 0.005
	− 64 − 30 + 14	L/R putamen	< 0.005
	+ 02 + 12 + 46	L/R insular cortex right	< 0.005
	+ 40 + 16 + 52	Superior frontal gyrus	< 0.005
	+ 02 + 12 + 46	Cerebellum (3–7)	< 0.005
IC20	− 18 + 30 + 60	Supplementary motor cortex	< 0.005
	− 58 − 18 + 32	R supramarginal gyrus	< 0.005
	− 60 − 20 + 30	L supramarginal gyrus	< 0.005
	+ 34 − 22 + 20	R hippocampus	< 0.005
	+ 48 + 04 + 26	R inferior frontal gyrus	< 0.005
	− 42 − 02 − 08	Insular cortex	< 0.005
	+ 34 − 22 + 20	L cerebellum (BA 4–5)	< 0.005
IC16	− 56 − 04 + 24	Supplementary motor cortex	< 0.005
	− 64 − 36 + 44	Supramarginal gyrus	< 0.005
	+ 18 + 18 − 12	L middle temporal gyrus	< 0.005
	+ 20 − 66 − 38	Cerebellum (BA 8)	< 0.005
	− 22 − 68 − 40	Cerebellum (BA 7)	< 0.005

Clustering indicates the spatial distribution of ROIs at the cortical and subcortical levels. Cluster threshold at p < 0.05 (pFDR corrected) and voxel threshold p < 0.001 (pFDR corrected, two sided). For each cluster we selected the brain regions with the highest covering proportion (%) of Harvard–Oxford Atlas ROI. Peak are reported in MNI coordinates.

*L* left, *R* right, *BA* Brodmann area.

## Discussion

ER processes are daily employed by individuals to modify the emotional states they are experiencing. However, the individuals’ variability to use one or other strategy is still poorly understood. How can such variability be explained? In the present study, we investigated the relationship between trait EI and ER abilities, on the basis of previous evidence that trait EI may represent a suitable measure for the individual predisposition to the choice of specific ER strategy (e.g.^18,25^). Namely, we were interested in finding the neural bases associated with trait EI by analysing the functional connectivity of naturally grouping circuits decomposed by an unsupervised machine learning approach (ICA). One intriguing question was whether EI and ER share at least some neural bases. This would be an additional proof of their intimate relationship.

In terms of trait measures, we found that individuals with low levels of trait EI are associated with the use of non-adaptive ER processes (i.e., suppression, and self-blame). This result is complementary to past evidence^18,25,67^ showing a relationship between high trait EI and adaptive coping strategies. The different, still complementary result can be explained by the fact that the previous studies (e.g.^25^) focused mostly on coping abilities and not on typical emotion regulation strategies. Instead, by the wide range of strategies considered, we found that low scores in the trait EI subscale of self-control are associated with blaming others and rumination, whereas low scores in the well-being subscale are associated with the use of suppression, rumination, and catastrophizing. Finally, low scores in emotionality are associated to the use of suppression and rumination.

In literature, rumination and suppression strategies have been usually related to negative outcomes and psychopathologies^5^. Likewise, low level of traits EI are also associated with psychopathological disorders^68,69^, making reasonable the strong link we found between low trait EI and maladaptive strategies. The specific association between rumination and self-control, which is associated with difficulties in managing stressful situations and impulsive behaviours, is supported by recent evidence suggesting that impulsivity plays a critical role in rumination^70^. Low self-esteem—a relevant aspect in the trait EI subscale of well-being, already emerged as an important predictor of rumination^71^, and it has been indirectly linked to suppression since it is involved in shame emotion, which has been already coupled with this regulation strategy^56,72^. In addition, low emotionality and the related difficulty in emotions recognition and expression^62^, may lead individuals to use suppression that occurs late in the process of emotion generation^73^. In our study, that trait EI subscale of sociability did not predict any emotion regulation strategy, can be explained by evidence showing that low sociability is not associated with indexes of emotion dysregulation (e.g., high psychological reactivity, negative emotional intensity, dispositional negative affect, and personal distress). Low sociability is rather associated with low social support seeking^74^, strategy which may have escaped the taxonomy used in the ERQ and CERQ. In support of that, trait EI predicts social sharing when this latter is included in the set of regulation strategies^28^. Our result also showed that women are more likely to use suppression with respect to men. Although also previous studies reported gender difference in the use of suppression^75–77^), this result is not consistent in literature (see^6,78^), suggesting that gender difference possibly depend on other factors such as the situation itself^79^ Further researches are needed to better understand the role of gender in the trait EI-ER relationship.

Besides trait measures, functional connectivity results showed that modulations of BOLD temporal variability in sensorimotor (IC20 and IC16), visual (IC18), salience (IC7) and cerebellar (IC1) networks is associated with the total traits EI index and the four subscales. Most importantly for the present study, we found that *increased* BOLD variability especially in sensorimotor (when identified by the IC20 but not IC16) and salience networks also predicted the use of reappraisal strategy (in ERQ and CERQ), whereas *decreased* BOLD variability in salience network predicted the use of emotion suppression. These results suggest that BOLD temporal variability in sensorimotor and salience networks underlies trait EI and at the same time explains differences in ER strategies. Namely, this finding enriches our understanding of the relationship between trait EI and ER, by showing for the first time that different networks involved in the former, are also involved in the latter.

The brain areas belonging to IC20 have been already related to the sensorimotor network^80^. Among these areas, the inferior frontal gyrus also emerged in previous resting-state study on trait EI^46^ and it was interpreted as a part of a circuit related to social and emotional processing. Similarly, a positive correlation between r-state connectivity involving frontal regions and trait EI was reported by Takeuchi et al.^48^. On the other hand, the supplementary motor cortex (SMA) and the cerebellum positively correlated with trait EI^46^, playing a critical role in cognitive control mechanisms. In particular, the somatosensory cortex, and its portion of the supramarginal gyrus (SMG) are involved in the recognition of emotions, the understanding of the emotional states of others^81^, and are more generally part of the mirror neuron system^82,83^. In addition, other studies provided evidence that the somatosensory cortex is associated with emotion generation^84^ and interoceptive awareness^85^. The insula, instead, is deemed to facilitate social interaction, and decision making by integrating sensory, affective, and bodily information, and it has been traditionally reported as neural correlate of trait EI. The implications of these areas in socio-emotional processing and cognitive control^46,86^ make intuitive their involvement also in reappraisal strategy, which involve cognitive abilities (e.g., attention and memory) to control emotional responses^87^. Consistently, there is evidence of a correlation between the frequency of use of reappraisal and the functional connectivity of the left insula, supplementary motor cortex (SMA)^49^, and inferior frontal gyrus^88^. These areas, more generally, represent a well-established network underlying cognitive control of emotions by reappraisal^34,38^. Our result concerning the negative correlation between the BOLD variability in the IC16-related sensorimotor network and the positive reappraisal seems apparently incongruent, then. However, this incongruency may be better interpreted in line with our trait measures results, which show no association between the trait EI-sociability scale and ER strategies. Similarly, indeed, that the IC16 (but not the IC20) is associated with the sociability scale may explain why this component yield a negative correlation with reappraisal. In addition, it is worth mentioning that the spatial match to template approach reports the IC20 (vs the IC16), as the best match for the sensorimotor network (see correlations in the Results section), and, yet, the two sensorimotor-related components involve overlapping, but still different brain regions, such as the middle temporal gyrus. Since this is the first attempt showing common neural substrates between trait EI and ER, further researchers are needed to better identify the specific involved brain areas.

Along the sensorimotor network, our study showed the involvement of the salience network, the BOLD variability of which positively correlates and predicts adaptive (reappraisal) strategy, and negatively correlates with maladaptive (suppression) strategy. In line with our results, previous studies reported the link between a similar component and the salience network^89^, as well as the relationship between the key nodes of this network and trait EI and ER^90^. Especially the amygdala and the basal ganglia (i.e., putamen), allowing the organism to adaptively respond to the emotional context, are strictly related to the emotion processing^91^ and as a such to the two constructs^48,49^. In addition, there is evidence that emotion suppression is associated with activation of anterior cingulate, ventrolateral prefrontal cortex^92^, inferior frontal gyrus, putamen, pre-supplementary motor area and supramarginal gyrus^93^.

The relation between BOLD variability, trait EI and adaptive vs maladaptive emotion regulation strategies, however, can be better understood coming back to what the BOLD variability means. Several studies point out that greater BOLD variability positively influences cognitive performance^94^, fluid intelligence^95^ and most importantly for our results, interoceptive awareness^96^, adaptability, flexibility and efficiency of neural system in response to the multiplicity and uncertainty of environmental stimuli^54,56,97,98^. As a such, it is reasonable that cognitive and affective mechanisms related to the functionally connected regions are better implemented by individuals showing increased temporal variability in the network^96^. Accordingly, our findings suggest that the increased BOLD variability in the sensorimotor and salience networks play a critical role in predicting both high level of trait EI and adaptive emotion regulation strategy, in terms of a better social and emotional information integration, self-awareness along with a more efficient cognitive control. That temporal variability in these networks significantly predicts high traits EI and the frequency of use of Cognitive Reappraisal strategy is explained as an adaptive feature of the neural response, allowing the brain to easily access different “states”, required to complete cognitive tasks^55,99^. By the same token, we could also infer that less variability in salience network could imply a difficulty of the subjects to process emotional information resulting in the maladaptive emotion suppression strategy. Then, greater temporal variability may represent a neural predisposition marker which facilitates individuals in the stages underlying the dynamic process of emotional regulation, identification, selection, and implementation^100^. This finding provides a context for and corroborates the hypothesis that regulation strategies and their outcome may depend on factors such as the individual differences. Neural flexibility and adaptability increase perception and control of the emotional event determining at the same time the success of an emotion regulation process^7^. Importantly, resting-state functional connectivity may be helpful to investigate task-independent constructs^101^ such as those related to personal traits.

Besides these new findings, the study has some limitations to point out. While the data-driven approach allowed us to consider the activity of the whole brain and the role of naturally grouping circuits, theory driven analyses (i.e., dynamic causal model) that may facilitate inferences with respect to specific brain regions, or to identify causal relationships between them, may be a valuable and complementary alternative. Moreover, we acknowledge that a larger sample size (including thousands of participants) would have been ideal for increasing reproducibility and statistical power, as a recent paper suggested^102^. For what concerns the discussed networks as identified by the spatial match, they also included portions of executive networks. Future studies are needed to explore the contributions of such networks to both EI and ER. Finally, building on the existing literature, we focused on trait EI. However, it would be worthy not only to investigate other aspects of EI^18^, but also to extend the knowledge on the interaction between affective mechanisms and personality, considering the variability this latter may show across different situations^103^.

To conclude, the present findings reveal that the role of trait EI in predicting adaptive ER strategies relies on a shared and more efficient functional connectivity network involved in social and emotional information processing to understand self and others’ affective states, and in higher cognitive mechanisms which contribute to the control of emotions. Consequently, our study not only further strengthened the association between low traits EI and maladaptive ER strategies but is also represents a first step to understand the neural mechanisms able to explain this relationship. Increased variability of the BOLD signal within a sensorimotor and salience network is a mainstay for the neural structure of high traits EI and at the same time predisposes to the use of adaptive emotional regulation strategy. By contrast, a decreased variability in salience network predisposed to the use of a maladaptive emotion regulation strategy.

## Methods

### Participants

The data analysed in this study were selected from the open-source dataset “Max Planck Institute Leipzig Mind-Brain-Body Dataset LEMON”^104^. All subjects were recruited by researchers at the University of Leipzig, in Germany, between 2013 and 2015, and data were collected in accordance with the Declaration of Helsinki for a study which protocol was approved by the ethics committee at the medical faculty of the University of Leipzig. For the present study, we extracted a subset of participants representing a young adult healthy population. Based on the information provided by the authors, indeed, we defined the sample by the following inclusion criteria: no substance use or abuse (negative at the Multi 8/2 Drogen-Tauchtest)^104^, and no past or present psychopathologies diagnosis, screened by the SCID-I, and by the Hamilton Depression Scale (HAM-D^105^). The final subset was composed of 79 subjects (23 females; age range: 20–35 years; mean education: 12.39 years). For each participant, we extracted raw data from structural MRI scans (T1 Weighted-MP2RAGE) and functional MRI scans (rs-fMRI). With regards to trait measures, the scores of the following self-administered questionnaires were selected: TeiQUE-SF (Trait EI Questionnaire-Short Form), ERQ (ER Questionnaire) and CERQ (Cognitive ER Questionnaire).

### Trait measures

The trait EI questionnaire short-form (TEIQue—SF) is used to measure EI as a personality trait^62^. The questionnaire was administered in the German validated version^106^ and assessed four factors: well-being (α = 0.94), self-control (α = 0.86), emotionality (α = 0.90), sociability (α = 0.88), plus a total index of trait EI, which consists of the average of the above factors (α = 0.96). As reported by the authors, reliability of the model-fit of the German validated version was acceptable to good: χ^2^ (54, N = 352) = 147.78, CFI = 0.95, SRMR = 0.049 and RMSEA = 0.07) and the test validity was reliable (0.88 ≤ α ≤ 0.96)^106^.

The emotion regulation questionnaire (ERQ) is adopted to measure the interindividual differences in the frequency of use of ER strategies^12^. The ERQ questionnaire was administered in the German validated version^107^, and consists of 10 items evaluating the tendency to use cognitive reappraisal (6 items, α = 0.73) and suppression of emotions (4 items, α = 0.76). The German version showed a good internal validity (0.73 ≤ α ≤ 0.76) and a good reliability (RMSEA = 0.068, CFI = 0.94, SRMR = 0.056)^107^.

The cognitive emotion regulation questionnaire (CERQ^108^) was administered in German validated version^63^ and consists of 36 items divided into 9 scales that measure nine strategies defined as adaptive: acceptance (α = 0.75), positive refocusing (α = 0.86), refocus on planning (α = 0.77), focus on positivity (α = 0.78), putting into perspective (α = 0.77); and four non-adaptive strategies: self-blame (α = 0.73), blaming others (α = 0.67), rumination (α = 0.66), catastrophizing (α = 0.73) . The German version of the scale showed good reliability (0.66 ≤ α ≤ 0.84) and a good model-fit factorial validity (χ2 = 482,267, df = 288, p < 0.01; RMSEA = 0.066; CFI = 0.922; TLI = 0.905)^63^ (see Table 4 for a summary of sample’s descriptive statistics for each scale and subscale).

**Table 4:** Descriptive statistics of TEIQue-SF, ERQ and CERQ questionnaires, included their subscales for N = 79.

Descriptive statistics				
	Score range		M	SD
	Min	Max		
ERQ-reappraisal	2.50	6.83	4.67	0.91
ERQ-suppression	1.25	5.75	3.64	1.12
CERQ-SelfBlame	0	11	4.05	2.25
CERQ-Acceptance	0	12	7.09	3.03
CERQ-Rumination	0	12	5.49	2.85
CERQ-positiveRefocusing	0	12	4.96	2.72
CERQ-RefocusOnPlanning	3	12	8.38	2.51
CERQ-PositiveReappraisal	0	12	7.00	2.68
CERQ-PuttingIntoPerspective	1	12	7.27	2.69
CERQ-Catastrophizing	0	10	2.04	2.16
CERQ-BlamingOthers	0	9	2.38	1.92
TEIQue-SF-totalEI	124	186	156.90	13.73
TeiQue-SF-selfControl	3.33	6.66	5.10	0.78
TeiQueSF-emotionality	3.50	6.75	5.16	0.76
TeiQueSF-sociability	3.33	6.50	5.08	0.62
TeiQueSF-well-being	3.00	7.00	5.87	0.75

*M* mean, *SD* standard deviation, *Min and Max* minimum and maximum scores for each subscale.

### MRI data acquisition

Structural and functional MRI data in the LEMON dataset were acquired with a 3 Tesla MRI scanner (Verio, Siemens Healthcare GmbH). During the acquisition, subjects were asked to remain awake with open eyes while looking at a low-contrast fixation cross. For our analyses we considered a BOLD rs-fMRI scan, using T2-weighted multiband EPI* sequence (TR = 1400 ms, TE = 30 ms, flip angle = 69°, echo spacing = 0.67 ms number of volumes = 657, voxel size = 2.3 mm, total acquisition time was 15 min 30 s) and T1-weighted structural volumes acquired using MP2RAGE sequence (TR = 5000 ms, TE = 2.92 ms, TI1 = 700 ms, TI2 = 2500 ms, FOV = 256 mm, voxel size = 1 mm isotropic) The structural volumes were acquired with 176 slices interspersed during 8 min 22 s of scanning^104^.

### Trait measures analyses

To test whether trait EI predicts ER processes, we implemented two different analyses using SPSS Statistics for Windows, version 25.0 (SPSS Inc., Chicago, Ill., USA). In the first analysis we implemented a multivariate linear regression (MLR) with ERQ and CERQ questionnaires as dependent variables, while the total trait EI was included as a predictor. Moreover, to assess the effect of every subscale of trait EI, we next implemented a multivariate multiple linear regression (MMLR) with each subscale of the ERQ and CERQ questionnaires as dependent variables, and the four factors of the TeiQue-SF as predictors. Variance inflation factor (VIF) and Pearson’s correlation among the TEIQue-SF subscales were used in order to examine multi-collinearity and relative association in the regression model. Gender was used as a categorical fixed factor to test its effect in the regression model. The type I error was controlled by applying false discovery rate (FDR) correction to p-values.

### Neuroimaging analyses

Pre-processing and functional connectivity analysis were conducted using CONN MATLAB Toolbox (version 18b)^109^. Firstly, we implemented CONN’s default pre-processing pipeline using SMP12 default parameters which includes the following steps: functional realignment and unwarping, translation and centering, functional outlier detection (conservative settings), functional direct segmentation and normalization (1 mm resolution), structural translation, and centering, structural segmentation and normalization (2.4 mm resolution), functional and structural smoothing (spatial convolution with Gaussian kernel 8 mm). Next, *the denoising* phase was implemented. The objective of this phase is the identification and elimination of confounding variables and artefacts from the estimated BOLD signal. Briefly, these factors are derived from three different sources (BOLD signal coming from white matter or cerebrospinal fluid masks, parameters and outliers defined in the pre-processing step, and an estimate of the pre-processing the subjects’ motion parameters)^110^. Once identified, the factors are entered into a regression model (Ordinary Least Squares) as covariates. Finally, a 0.0008–0.09 Hz temporal band-pass filter standard for resting-state connectivity analyses was applied to the time series. Next, the functional connectivity analysis has been implemented. For this study, we chose to use a data-driven approach by implementing a group-independent component analysis (group-ICA). The group-ICA implemented by CONN includes the following steps: pre-conditioning variance normalization, concatenation of the BOLD signal along the temporal dimension, dimensionality reduction at the group level, fast-ICA for spatial component estimation, and the back-projection for spatial estimation on the individual subject^110^. The number of independent components to be identified was set to 20 as software CONN suggests as default, and in line with previous studies using low model order analysis^111–113^. In order to separate noise components from the underlying resting-state networks, every identified IC were visually inspected and compared with CONN’s networks atlas using spatial match-to-template function. This feature measures the overlap between eight brain networks (Default Mode Network, Sensorimotor, Visual, Salience, Dorsal Attention, Frontoparietal, Language, Cerebellar; defined from CONN’s ICA analyses of HCP dataset/497 subjects) and the IC’s spatial map associated with each individual network component. One out of 20 ICs (IC17) did not allow for the delineation of specific areas due to its extent and it was discarded from the following analyses. We then extracted the temporal variability of each remaining IC’s, calculated in CONN as SD of each BOLD time-series^110^. Type I error was controlled using cluster-size-based false discovery rate (FDR) correction (*p* < 0.05, voxel thresholded at *p* < 0.001^114^ tab, within each analysis). Next, to assess the relationship between IC’s temporal variability and both trait EI and ER, we implemented 2 different analysis by using SPSS Statistics for Windows, version 25.0 (SPSS Inc., Chicago, Ill., USA). Firstly, to address which of the 20 identified IC’s predicted the trait EI, we tested the individual explanatory variables effect (IC’s BOLD variability values) on the TEIQue-SF factors and total index by using a Multiple Linear Regression model (Ordinary Least Squares) with a stepwise method (forward) for each dependent variable, and gender as a categorical fixed factor in order to test its effect in the regression model. Since we do not expect that all the identified components were related to the investigated construct, we chose a method of fitting regression models in which the choice of predictor variables is made by an automatic procedure. This methodology consists of testing the incremental predictivity of the model: starting from a model with no predictor, each explanatory variable is added to the model and compared to the inclusion or exclusion threshold criterion (in our case predictor’s p-value ≤ 0.05 for inclusion) until the model reaches its maximum predictivity. Finally, the BOLD temporal variability of IC’s that resulted to be significant predictors of trait EI in the previous analysis were entered a Multivariate Multiple Regression (MMR) as independent variables to predict ER scores (ERQ and CERQ subscales) and gender as a categorical fixed factor in order to test its effect in the regression model. To avoid multiple comparisons issues, type I error was controlled applying false discovery rate correction (FDR) within each analysis.

## Data Availability

The complete LEMON Data can be accessed via Gesellschaft für wissenschaftliche Datenverarbeitung mbH Göttingen (GWDG) https://www.gwdg.de/. Raw and preprocessed data at this location is accessible through web browser https://ftp.gwdg.de/pub/misc/MPI-Leipzig_Mind-Brain-Body-LEMON/ and a fast FTP connection (ftp://ftp.gwdg.de/pub/misc/MPI-Leipzig_Mind-Brain-Body-LEMON/). In the case the location of the data changes in the future, the location of the dataset can be resolved with PID 21.11101/0000-0007-C379-5 (e.g. http://hdl.handle.net/21.11101/0000-0007-C379-5).
